# Cardiovascular Events in Patients with Atherothrombotic Disease: A Population-Based Longitudinal Study in Taiwan

**DOI:** 10.1371/journal.pone.0092577

**Published:** 2014-03-19

**Authors:** Wen-Hsien Lee, Po-Chao Hsu, Chun-Yuan Chu, Ho-Ming Su, Chee-Siong Lee, Hsueh-Wei Yen, Tsung-Hsien Lin, Wen-Chol Voon, Wen-Ter Lai, Sheng-Hsiung Sheu

**Affiliations:** 1 Division of Cardiology, Department of Internal Medicine, Kaohsiung Medical University Hospital, Kaohsiung Medical University, Kaohsiung, Taiwan; 2 Department of Internal Medicine, Kaohsiung Municipal Hsiao-Kang Hospital, Kaohsiung Medical University, Kaohsiung, Taiwan; 3 Faculty of Medicine, College of Medicine, Kaohsiung Medical University, Kaohsiung, Taiwan; Shanghai Institute of Hypertension, China

## Abstract

**Background:**

Atherothrombotic diseases including cerebrovascular disease (CVD), coronary artery disease (CAD), and peripheral arterial disease (PAD), contribute to the major causes of death in the world. Although several studies showed the association between polyvascular disease and poor cardiovascular (CV) outcomes in Asian population, there was no large-scale study to validate this relationship in this population.

**Methods and Results:**

This retrospective cohort study included patients with a diagnosis of CVD, CAD, or PAD from the database contained in the Taiwan National Health Insurance Bureau during 2001–2004. A total of 19954 patients were enrolled in this study. The atherothrombotic disease score was defined according to the number of atherothrombotic disease. The study endpoints included acute coronary syndrome (ACS), all strokes, vascular procedures, in hospital mortality, and so on. The event rate of ischemic stroke (18.2%) was higher than that of acute myocardial infarction (5.7%) in our patients (P = 0.0006). In the multivariate Cox regression analyses, the adjusted hazard ratios (HRs) of each increment of atherothrombotic disease score in predicting ACS, all strokes, vascular procedures, and in hospital mortality were 1.41, 1.66, 1.30, and 1.14, respectively (P≦0.0169).

**Conclusions:**

This large population-based longitudinal study in patients with atherothrombotic disease demonstrated the risk of subsequent ischemic stroke was higher than that of subsequent AMI. In addition, the subsequent adverse CV events including ACS, all stroke, vascular procedures, and in hospital mortality were progressively increased as the increase of atherothrombotic disease score.

## Introduction

Atherothrombotic diseases including cerebrovascular disease (CVD), coronary artery disease (CAD), and peripheral arterial disease (PAD), contribute to the major causes of death in the world. Meanwhile, in Taiwan, the CAD and CVD rank as the second and third leading causes of death and possess a great budget on healthcare system in recent years [1]–[3]. In the future, the atherothrombotic disease will still be predicted as the leading cause of death worldwide by 2020 [4].

The REduction of Atherothrombosis for Continued Health (REACH) Registry was an international and observational study which enrolled patients with established atherosclerotic arterial disease or multiple risk factors of atherothrombosis and evaluated the medical management and risks of cardiovascular (CV) events in these patients [5]. It demonstrated patients with polyvascular disease were associated with a significantly higher risk of adverse CV events [6]–[8]. Although the REACH Registry included 69,055 patients, there were only 13.5% patients enrolled from the Asia. In patients with atherothrombotic disease, the most occurred CV event was CAD in Western population, but was CVD in Asian population [9]–[12]. Although several studies showed the association between polyvascular disease and poor CV outcomes in Asian population [13], [14], there was no large-scale study to evaluate the relationship between polyvascular disease and adverse CV events in Taiwanese. Therefore, we conducted this large study to assess whether patients with polyvascular disease were independently associated with increased CV events in Taiwanese.

## Methods

### Ethics statement

The retrospective study protocol was approved by the institutional review board of the Kaohsiung Medical University Hospital (KMUH-IRB-EXEMPT-20130049). Because patient records and information was anonymized and de-identified prior to analysis, the written informed patient consent was waived by the IRB.

### Data source

The data was analyzed from the National Health Insurance Research Dataset (NHIRD), published by the National Health Research Institute (NHI) in Taiwan, which provided a database of 1,000,000 random subjects. The NHI program has been implemented in Taiwan since 1995, offering a comprehensive, unified, and universal health insurance program to all citizens. All citizens who have established a registered domicile for at least 4 months in the Taiwan area should be enrolled in NHI. The coverage provides outpatient service, inpatient care, Chinese medicine, dental care, childbirth, physical therapy, preventive health care, home care, and rehabilitation for chronic mental illness. The coverage rate was 96% of whole population in 2000 and was elevated to 99% at the end of 2004. The NHI medical claim database included ambulatory care, hospital inpatient care, dental services, and prescription drugs. Therefore, the NHIRD is one of the largest and most complete nationwide population-based datasets in Taiwan and there were no statistically significant differences in age, sex, and average insured payroll-related amount between the sample group and all enrollees.

### Study sample

This study population consisted of all patients with more than two-time outpatient or inpatient claims with a diagnosis of CVD, CAD, or PAD between July 1, 2001 and December 31, 2004. Initially, these patients with CVD, CAD, or PAD were enrolled by their disease code of international classification of diseases 9^th^ revision clinical modification (ICD-9-CM) or their vascular location related procedure codes of ICD-9-CM during study period [15]-[18]. Therefore, the patients with CVD were enrolled by ICD-9-CM (433–438) or its related procedures, such as carotid angioplasty and carotid endarterectomy (procedure codes: 00.61, 00.63, 38.1). The patients with CAD were enrolled by ICD-9-CM (410–414) or its related procedures, such as coronary angioplasty/stenting and coronary artery bypass grafting (procedure codes: 36.01–36.02, 36.05 to 36.07, 36.10–36.20). The patients with PAD were enrolled by ICD-9-CM (250.7, 443, 443.81, 443.9, 785.4, and 444.2) or its related procedures, such as peripheral angioplasty and lower extremity amputation (procedure codes: 84.1, 84.10-84.18). Then, the data of the first claim with a CVD, CAD or PAD diagnosis was considered the index date. The patients who were younger than 45 years old and had a diagnosis of CVD, CAD or PAD before index date were excluded. Finally, these patients with newly diagnosed CVD, CAD or PAD who received any antithrombotic or antiplatelet agents, such as aspirin, clopidogrel, ticlopidine, warfarin, or cilostazol, ≧ 30 days after each patient's index date were finally identified to form our study patients [19]. Based on these claim data, the patients were then classified according to the number or location of vascular disease. Baseline characteristics including age, sex, treatment information, and comorbidities were extracted from all claims within 180 days before the index data. Baseline comorbidities included diabetes mellitus (DM) (ICD-9-CM: 250), hypertension (ICD-9-CM: 401–405), hyperlipidemia (ICD-9-CM: 272.0–272.4), congestive heart failure (ICD-9-CM: 428), atrial fibrillation (ICD-9-CM: 427.31), chronic obstructive pulmonary disease (ICD-9-CM: 491, 492, 496), chronic kidney disease (ICD-9-CM: 585), aortic and mitral valve stenosis (ICD-9-CM: 394–396), peptic ulcer disease (ICD-9-CM: 531–534), monthly income (New Taiwan [NT] $0, NT $1-20000, NT $>20000), using of anti-thrombotic agents (aspirin, clopidogrel, ticlopidine, warfarin, and cilostazol), using of anti-hypertensive agents (angiotensin converting enzymes, angiotensin II receptor blockers, β blockers, calcium channel blockers, diuretics, hydralazine, α blockers, and central α-2 adrenergic agonist), using of anti-diabetic agents (sulfonylureas, meglitinide, biguanides, thiazolidinediones, acarbose, and insulins), using of lipid-lowing agents (statins, fibrates, acipimox, and cholestyramine), using of proton pump inhibitors, urbanization level (ranging from most urbanized [level 1] to least urbanized level [level 5]), and hospital level (including medical center, regional hospital, district hospital and clinics). Patients with the single vascular disease were defined as those with CVD, CAD, or PAD. Patients with the double vascular disease were defined as those with CVD and CAD, CVD and PAD, or CAD and PAD. Patients with the triple vascular disease were defined as those with CVD, CAD, and PAD.

### Study outcomes

The study endpoints included acute myocardial infarction (AMI) (ICD-9-CM: 410), unstable angina (ICD-9-CM: 411.1), acute coronary syndrome (ACS) including AMI and unstable angina, hemorrhagic stroke (ICD-9-CM: 430–432), ischemic stroke (ICD-9-CM: 433–436), other strokes including undetermined types of stroke and stroke sequela (ICD-9-CM: 437–438), all strokes including ischemic, hemorrhagic, and other strokes, vascular procedures, and in hospital mortality. The vascular procedures included carotid angioplasty and endarterectomy, coronary angioplasty/stenting and coronary artery bypass grafting surgery, and peripheral angioplasty and lower extremity amputation. In patients reaching the study endpoints, they were followed until the first episode of adverse CV events. The other patients were followed until December 2008.

### Statistical analysis

Categorical variables among groups were compared by Chi-square analysis. Continuous variables among groups were compared by one-way analysis of variance. Time to CV events was assessed by Cox regression analysis. The atherothrombotic disease score was defined according to the number of atherothrombotic disease, i.e. 1, 2, and 3 points were defined for single, double, and triple vascular diseases, respectively. Gender and significant variables in the univariate analysis were selected into the multivariate Cox regression analysis. Significance was set at p<0.05. All the data processing and statistical analyses were performed with SAS 9.3 software.

## Results

**Figure 1:**
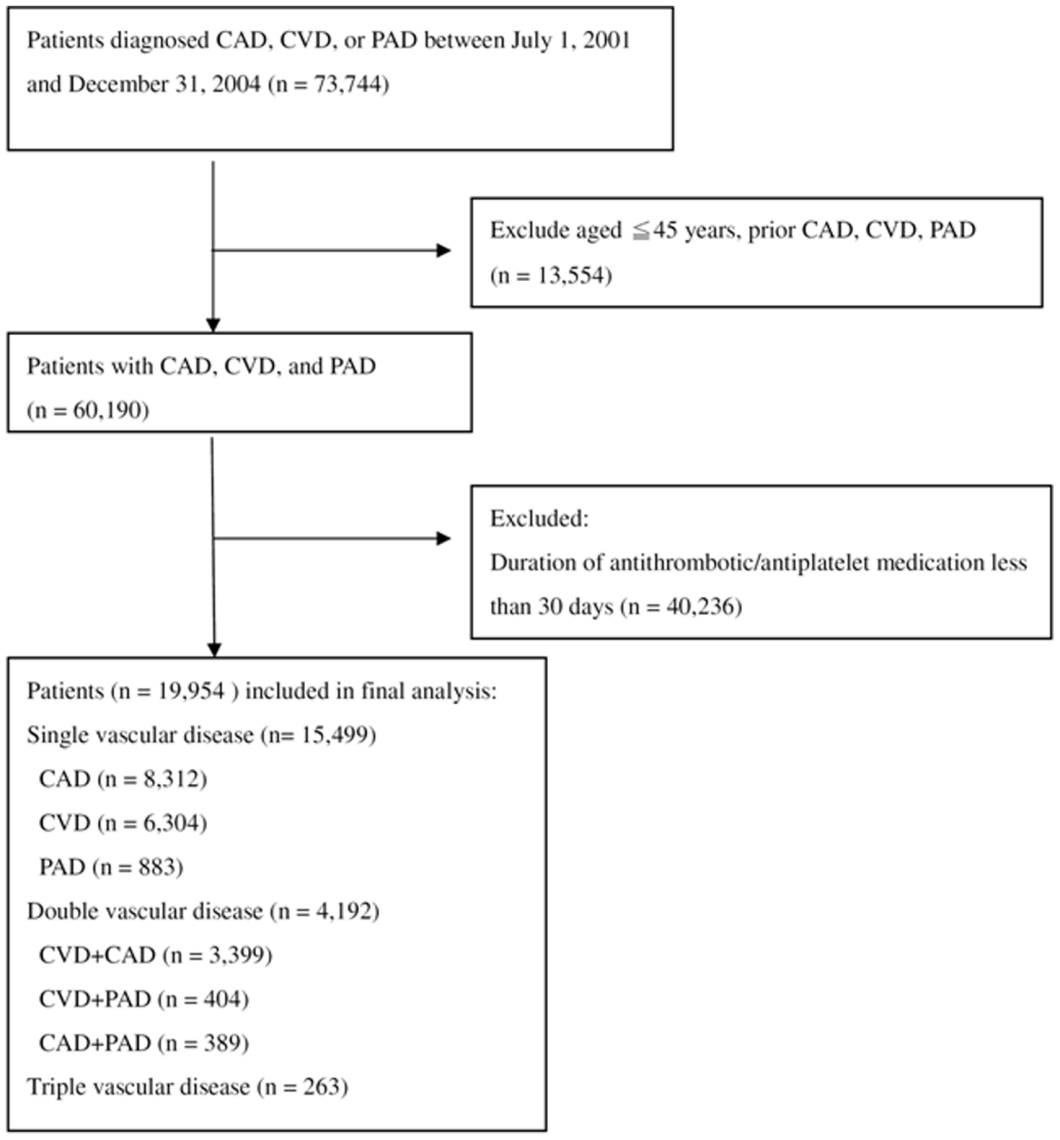
Flow chart of study participants. CVD, cerebrovascular disease; CAD, coronary artery disease; PAD, peripheral arterial disease.

Of the 73,744 patients diagnosed as CVD, CAD or PAD from January 2001 to December 2004, 19,954 patients (53.5% male) met all the inclusion criteria (Fig. 1). The mean follow-up period was 1198±8 days. Table 1 shows the comparison of baseline characteristics in patients with single, double, and triple vascular diseases. There were significant differences in age, the prevalence of DM, hypertension, hyperlipidemia, atrial fibrillation, chronic obstructive pulmonary disease, and peptic ulcer disease, using of anti-hypertensive agents, anti-diabetic agents, lipid-lowing agents, and proton pump inhibitors, monthly income, and hospital level in patients with single, double, and triple vascular diseases.

**Table 1:** Comparison of baseline characteristics in patients with single, double, and triple vascular diseases.

Variables/Groups, N (%)	Total patients	Single vascular disease	Double vascular disease	Triple vascular disease	P value
	N = 19954	N = 15499 (77.7)	N = 4192 (21.0)	N = 263 (1.3)	
Gender (male)	10666 (53.5)	8286 (53.5)	2233 (53.3)	147 (55.9)	0.7091
Age (mean ± SD)	66.05±10.32	65.71±10.46	67.16±9.75	68.27±9.42	<0.0001
Age ≧65 years old	11465 (57.5)	8660 (55.9)	2628 (62.7)	177 (67.3)	<0.0001
Diabetes mellitus	5569 (29.9)	4203 (27.1)	1262 (30.1)	104 (39.5)	<0.0001
Hypertension	12481 (62.5)	9613 (62.0)	2687 (64.1)	181 (68.8)	0.0051
Hyperlipidema	4171 (20.9)	3209 (20.7)	889 (21.2)	73 (27.8)	0.0176
Congestive heart failure	1360 (6.8)	1035 (6.7)	303 (7.2)	22 (8.4)	0.2753
Atrial fibrillation	448 (2.2)	330 (2.1)	115 (2.7)	3 (1.1)	0.0280
Chronic kidney disease	608 (3.0)	448 (2.9)	151 (3.6)	9 (3.4)	0.0555
Chronic obstructive pulmonary disease	2393 (14.7)	2199 (14.2)	684 (16.3)	50 (19.0)	0.0004
Aortic and mitral valve stenosis	291 (1.5)	238 (1.5)	49 (1.2)	4 (1.5)	0.2128
Peptic ulcer disease	4114 (20.6)	3122 (20.1)	937 (22.4)	55 (20.9)	0.0073
**Medication**					
Anti-thrombotic agents	19889(99.7)	15449(99.7)	4178(99.7)	262(99.6)	0.9814
Anti-hypertensive agents	18870 (94.6)	14559 (93.9)	4056 (96.8)	255 (97.0)	<0.0001
Anti-diabetic agents	7111 (35.6)	5360 (34.6)	1617 (38.6)	134 (51.0)	<0.0001
Lipid-lowering agents	9550 (47.9)	7306 (47.1)	2104 (50.2)	140 (53.2)	<0.0001
Proton pump inhibitors	6979 (35.0)	5169 (33.4)	1678 (40.0)	132 (50.2)	<0.0001
**Monthly income**					0.0008
0	6851 (34.3)	5300 (34.2)	1466 (35.0)	86 (32.7)	
NT$ 1-20000	10894 (54.6)	8407 (54.2)	2331 (55.6)	156 (59.3)	
NT$ >20000	2208 (11.1)	1792 (11.6)	395 (9.4)	21 (8.0)	
**Urbanization level**					0.1544
1	4379 (21.9)	3452 (22.3)	874 (20.8)	53 (20.2)	
2	1434 (7.2)	1145 (7.4)	271 (6.5)	19 (7.2)	
3	839 (4.2)	665 (4.3)	164 (3.9)	10 (3.8)	
4	8842 (44.3)	6800 (43.9)	1927 (46.0)	115 (43.7)	
5	4411 (22.1)	3398 (21.9)	947 (22.6)	66 (25.1)	
**Hospital level**					0.0480
Medical center	1189 (6.0)	908 (5.9)	261 (6.2)	20 (7.6)	
Regional hospital	1127 (5.6)	859 (5.5)	254 (6.1)	14 (5.3)	
District hospital	3787 (19.0)	2885 (18.6)	843 (20.1)	59 (22.4)	
Clinics	13851 (69.4)	10847 (70.0)	2834 (67.6)	170 (64.6)	

Abbreviations: NT, new Taiwan; SD, standard deviation. Urbanization level is ranging from the most urbanized (level 1) to the least urbanized level (level 5).

**Figure 2:**
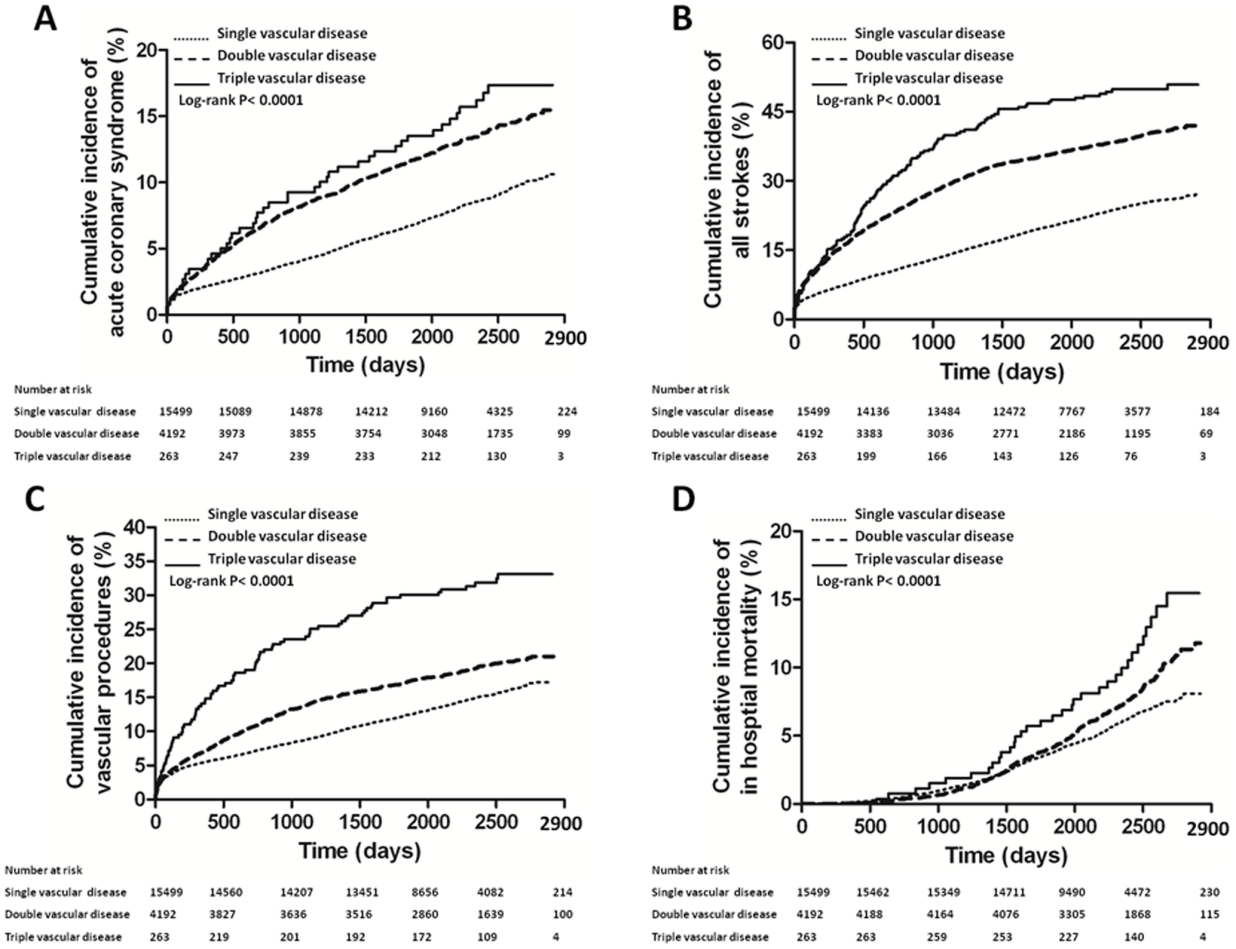
Kaplan-Meier cumulative risk curves for adverse cardiovascular events in patients with single, double, and triple vascular diseases. Kaplan-Meier cumulative risk curves for acute coronary syndrome (A), all strokes (B), vascular procedures (C), and in hospital mortality (D) in patients with single, double, and triple vascular diseases. All strokes included ischemic, hemorrhagic, and other strokes. Vascular procedures included carotid angioplasty and endarterectomy, coronary angioplasty/stenting and coronary artery bypass grafting surgery, and peripheral angioplasty and lower extremity amputation.

Table 2 shows the hazard ratios (HRs) of each increment of atherothrombotic disease score in predicting adverse CV events in all patients. The event rates of all strokes (26.5%) and ischemic stroke (18.2%) were higher than those of ACS (9.3%) and AMI (5.7%) in all study patients (P≦0.0028). After adjustment for gender and significant variables in the univariate analysis, the adjusted HRs of each increment of atherothrombotic disease score in predicting ACS, all strokes, vascular procedures, and in hospital mortality were 1.41, 1.66, 1.30, and 1.14, respectively (P≦0.0169). Figure 2 demonstrates the Kaplan-Meier cumulative risk curves for the ACS, all strokes, vascular procedures, and in hospital mortality in patients with single, double, and triple vascular diseases. There were significant differences in these four CV outcomes in patients with single, double, and triple vascular diseases (all Log-rank P<0.0001) (Figure 2A to 2D).

**Table 2:** The HRs of each increment of atherothrombotic disease score in predicting adverse cardiovascular events in all patients.

Events	Number (%)	Unadjusted HR (95%CI)	P value	Adjusted HR (95%CI)	P value
**Acute coronary syndrome**	1857 (9.3)	1.54 (1.41–1.68)	<0.0001	1.41(1.30–1.54)	<0.0001
AMI	1134 (5.7)	1.43(1.28–1.59)	<0.0001	1.30(1.16–1.45)	<0.0001
Unstable angina	1173 (5.9)	1.13(1.08–1.19)	<0.0001	1.06(1.01–1.12)	0.0123
**All strokes**	5297 (26.5)	1.78(1.70–1.87)	<0.0001	1.66(1.58–1.74)	<0.0001
Hemorrhagic stroke	837 (4.2)	1.76(1.56–1.99)	<0.0001	1.65(1.46–1.86)	<0.0001
Ischemic stroke	3640 (18.2)	1.71(1.61–1.81)	<0.0001	1.58(1.48–1.68)	<0.0001
Other stroke	3200 (16.0)	1.75 (1.64–1.86)	<0.0001	1.62(1.52–1.72)	<0.0001
**Vascular procedures**	3106 (15.6)	1.40 (1.31–1.50)	<0.0001	1.30(1.21–1.39)	<0.0001
**In hospital mortality**	1218 (6.1)	1.32(1.19–1.47)	<0.0001	1.14 (1.02–1.27)	0.0169

Abbreviations: AMI, acute myocardial infarction; CI, confidence interval; HR, hazard ratio.

Other strokes included undetermined types of stroke and stroke sequel.

In above multivariate Cox regression models, covariates included gender and the significant variables in the univariate analysis (age, diabetes, hypertension, hyperlipidemia, atrial fibrillation, chronic obstructive pulmonary disease, peptic ulcer disease, using of anti-hypertensive agents, anti-diabetic agents, lipid-lowing agents, and proton pump inhibitors, monthly income, and hospital level.).

We also performed subgroup analyses in patients with age between 45 and 64 years old and age ≧ 65 years old and in patients with different gender. Table 3 shows the adjusted HRs of each increment of atherothrombotic disease score in predicting adverse CV events in subgroup patients. In female patients, the adjusted HRs of each increment of atherothrombotic disease score in predicting ACS, all strokes, and vascular procedures were 1.53, 1.67, and 1.41, respectively (P≦0.0001). In male patients, the adjusted HRs of each increment of atherothrombotic disease score in predicting ACS, all strokes, vascular procedures, and in hospital mortality were 1.35, 1.66, 1.26, and 1.23, respectively (P≦0.0033). In patients with age between 45 and 64 years old, the adjusted HRs of each increment of atherothrombotic disease score predicting ACS, all strokes, and vascular procedures were 1.21, 1.84, and 1.20, respectively (P≦0.0160). In patients with age ≧ 65 years old, the adjusted HRs of each increment of atherothrombotic disease score in predicting ACS, all strokes, vascular procedures, and in hospital mortality were 1.53, 1.59, 1.36, and 1.13, respectively (P≦0.0384).

**Table 3:** The adjusted HRs of each increment of atherothrombotic disease score in predicting adverse cardiovascular events in subgroup patients.

	Female, number = 9288		Male, number = 10666		Age between 45-64 years old, number = 8489		Age ≧ 65 years old, number = 11465	
Events	Adjusted HR (95%CI)	P value	Adjusted HR (95%CI)	P value	Adjusted HR (95%CI)	P value	Adjusted HR (95%CI)	P value
**Acute coronary syndrome**	1.53(1.34–1.75)	0.0001	1.35(1.21–1.51)	<0.0001	1.21(1.04–1.40)	0.0160	1.53(1.38–1.70)	<0.0001
AMI	1.52(1.28–1.82)	<0.0001	1.19(1.03–1.38)	0.0179	1.10(0.91–1.36)	0.3085	1.41(1.23–1.62)	<0.0001
Unstable angina	1.07(0.99–1.15)	0.0769	1.06(1.00–1.13)	0.0685	1.12(1.03–1.22)	0.0088	1.04(0.98–1.11)	0.1671
**All strokes**	1.67(1.54–1.80)	<0.0001	1.66(1.55–1.77)	<0.0001	1.84(1.68–2.02)	<0.0001	1.59(1.50–1.69)	<0.0001
Hemorrhagic stroke	1.64(1.34–2.01)	<0.0001	1.65(1.41–1.92)	<0.0001	2.01(1.64–2.47)	<0.0001	1.49(1.28–1.73)	<0.0001
Ischemic stroke	1.56(1.42–1.72)	<0.0001	1.59(1.47–1.72)	<0.0001	1.78(1.61–1.98)	<0.0001	1.49(1.39–1.60)	<0.0001
Other stroke	1.62(1.47–1.79)	<0.0001	1.62(1.49–1.76)	<0.0001	1.85(1.64–2.08)	<0.0001	1.54(1.42–1.66)	<0.0001
**Vascular procedures**	1.41(1.26–1.59)	<0.0001	1.26(1.15–1.37)	<0.0001	1.20(1.07–1.34)	0.0016	1.36(1.25–1.49)	<0.0001
**In hospital mortality**	1.03(0.86–1.23)	0.7766	1.23(1.07–1.40)	0.0033	1.18(0.91–1.53)	0.2091	1.13(1.01–1.28)	0.0384

Abbreviations: AMI, acute myocardial infarction; CI, confidence interval; HR, hazard ratio.

Other strokes included undetermined types of stroke and stroke sequel.

In multivariate Cox regression models, covariates included gender and the significant variables in the univariate analysis.

## Discussion

In the relatively large population-based longitudinal study in patients with atherothrombotic disease, we demonstrated that the risk of subsequent ischemic stroke was higher than that of subsequent AMI. In addition, the subsequent adverse CV events including ACS, all stroke, vascular procedures, and in hospital mortality were progressively increased as the increase of atherothrombotic disease score.

Previous studies showed that stroke mortality and incidence were higher in Asian population than in Western population [9], [11]. In the REACH Registry, the ethnic comparison of 1-year CV outcomes showed that the event rate of non-fatal myocardial infarction was higher in North American (1.29%) than in Asia (0.82%), but the event rate of non-fatal stroke was higher in Asian (2.60%) than in North American (1.18%) [5]. A large-scale project, the Asia Pacific Cohort Studies Collaboration (APCSC), which included 44 separate cohorts and data from over 650,000 individuals, showed that hypertension, smoking, and DM were the major risk factors for fatal and non-fatal stroke [20]–[23]. The high event rate of stroke in Asian countries may be explained by a high prevalence of hypertension, low level of serum total cholesterol, and low proportion of effective risk-reducing treatment [9], [11]. Our present study similarly showed that the event rate of ischemic stroke was higher than that of AMI in Taiwanese patients with atherothrombotic disease.

In the REACH Registry, all major CV event rates increased with the number of vascular disease, ranging from 12.6% for patients with single, 21.1% for patients with double, and 26.3% for patients with triple vascular disease during 1-year follow up [6]. Furthermore, among patients with atherothrombosis, those with a prior history of myocardial infarction or stroke at baseline had the higher rate of subsequent ischemic events (18.3%) than those with a stable CAD, CVD, or PAD at baseline (12.2%) during 4-year follow up [7]. In the present study, in Taiwanese patients with atherothrombotic disease, we consistently found the subsequent adverse CV events including ACS, all stroke, vascular procedures, and in hospital mortality were progressively increased as the increase of atherothrombotic disease score.

Several reasons may illustrate that polyvascular disease is a strong predictor for future CV events. Although CAD, CVD, and PAD shared common risk factors, poor controlled risk factors including current smoking, high blood pressure, fasting glucose, and total cholesterol were more frequently in patients with polyvascular diseases than those with single vascular disease [24]. The ACS patients with prior atherothrombotic disease had higher in hospital mortality than those without prior atherothrombotic disease [25], [26]. In addition, inflammation may be an important factor for the initiation, progression, and linkage among CAD, CVD, and PAD [27], [28]. The inflammation triggered from the affected arterial bed may activate the endothelium at distant arterial beds. Patients with polyvascular diseases were reported to have higher levels/concentrations of inflammation makers, such as high sensitivity C reactive protein, neutrophil myeloperoxidase content, interleukin 6, intercellular adhesion molecule 1, vascular cell adhesion molecule 1, matrix metalloproteinase 9, cellular fibronectin, and so on [27]–[29]. In the present study, we similarly demonstrated polyvascular disease was significantly associated with an increase in future adverse CV events.

There were several limitations in our population-based longitudinal investigation. First, the database provided by NHI might have possible disease misclassifications and inadequate diagnostic codes for increasing payment to hospitals and administration of certain medications [30]. Second, we did not know the real data of ankle-brachial index, carotid intima-media thickness, National Institutes of Health Stroke Scale, and modified Rankin Scale. The education status, personal history of smoking and alcohol habits, and body mass index were also lacking in the NHI database. These factors might affect the subsequent CV events [13], [14], [31], [32]. Additionally, we did not know the severity of vascular diseases and devices of vascular intervention, which might also affect the subsequent CV outcomes [33]–[35]. Third, we did not enroll patients who were younger than 45 years old. In young patients, the etiologies of premature CAD or CVD were more heterogeneous than those in older patients [36]–[37]. Therefore, our results can not be applied in young patients with atherothrombotic disease. Finally, despite of the population based database, this study did not have any randomization which might have some selection bias.

## Conclusion

This large population-based longitudinal study in patients with atherothrombotic disease demonstrated the risk of subsequent ischemic stroke was higher than that of subsequent AMI. In addition, the subsequent adverse CV events including ACS, all stroke, vascular procedures, and in hospital mortality were progressively increased as the increase of atherothrombotic disease score.
